# Delayed intramuscular human neurotrophin-3 improves recovery in adult and elderly rats after stroke

**DOI:** 10.1093/brain/awv341

**Published:** 2015-11-27

**Authors:** Denise A. Duricki, Thomas H. Hutson, Claudia Kathe, Sara Soleman, Daniel Gonzalez-Carter, Jeffrey C. Petruska, H. David Shine, Qin Chen, Tobias C. Wood, Michel Bernanos, Diana Cash, Steven C. R. Williams, Fred H. Gage, Lawrence D. F. Moon

**Affiliations:** ^1^ 1 Neurorestoration Group, Wolfson Centre for Age-Related Diseases, King’s College London, 16–18 Newcomen Street, London SE1 1UL, UK; ^2^ 2 Centre for Integrative Biology, King’s College London, Franklin-Wilkins Building, 150 Stamford Street, London SE1 9NH, UK; ^3^ 3 Division of Brain Sciences, Department of Medicine, Hammersmith Campus, Imperial College London, London, UK; ^4^ 4 John Van Geest Centre for Brain Repair University of Cambridge, The E.D. Adrian Building, Forvie Site, Robinson Way Cambridge, CB2 0PY, UK; ^5^ 5 Department of Anatomical Sciences and Neurobiology, University of Louisville; Kentucky Spinal Cord Injury Research Center, Department of Neurological Surgery, Louisville, Kentucky, USA; ^6^ 6 Center for Cell and Gene Therapy, Department of Neuroscience, Alkek Bldg N1130.01, Baylor College of Medicine, One Baylor Plaza, Houston, Texas 77030, USA; ^7^ 7 Neuroimaging Research Group, King’s College London, PO42 De Crespigny Park, London, SE5 8AF, UK; ^8^ 8 The Salk Institute for Biological Studies, 10010 N. Torrey Pines Road, La Jolla, CA 92037, USA

**Keywords:** neurotrophin-3, stroke, plasticity, corticospinal, sprouting

## Abstract

There is an urgent need for a therapy that reverses disability after stroke when initiated in a time frame suitable for the majority of new victims. We show here that intramuscular delivery of neurotrophin-3 (NT3, encoded by *NTF3* ) can induce sensorimotor recovery when treatment is initiated 24 h after stroke. Specifically, in two randomized, blinded preclinical trials, we show improved sensory and locomotor function in adult (6 months) and elderly (18 months) rats treated 24 h following cortical ischaemic stroke with human NT3 delivered using a clinically approved serotype of adeno-associated viral vector (AAV1). Importantly, AAV1-hNT3 was given in a clinically-feasible timeframe using a straightforward, targeted route (injections into disabled forelimb muscles). Magnetic resonance imaging and histology showed that recovery was not due to neuroprotection, as expected given the delayed treatment. Rather, treatment caused corticospinal axons from the less affected hemisphere to sprout in the spinal cord. This treatment is the first gene therapy that reverses disability after stroke when administered intramuscularly in an elderly body. Importantly, phase I and II clinical trials by others show that repeated, peripherally administered high doses of recombinant NT3 are safe and well tolerated in humans with other conditions. This paves the way for NT3 as a therapy for stroke.

## Teaser

Duricki *et al.* show that intramuscular delivery of human neurotrophin-3 induces corticospinal plasticity and locomotor recovery in adult and elderly rats 24 hours post-stroke. This time-frame would be clinically feasible for most stroke victims, and the safety and tolerability of neurotrophin-3 in humans have been established for other disorders.

## Graphical abstract

**Figure:**
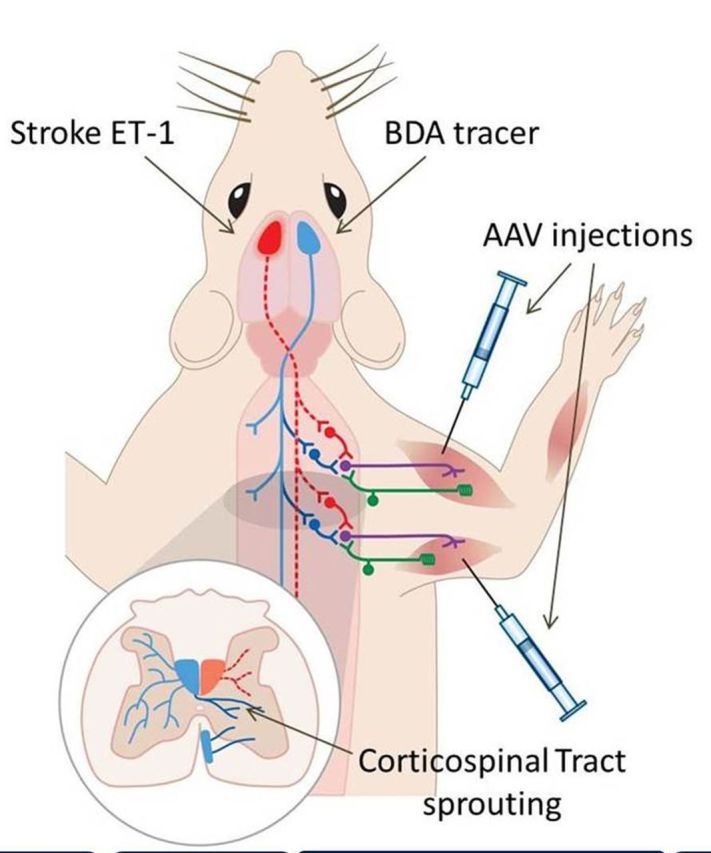
Duricki *et al.* show that intramuscular delivery of human neurotrophin-3 induces corticospinal plasticity and locomotor recovery in adult and elderly rats 24 hours post-stroke. This time-frame would be clinically feasible for most stroke victims, and the safety and tolerability of neurotrophin-3 in humans have been established for other disorders.

## Introduction

Stroke rapidly kills brain cells and is frequently disabling. Globally, there are 31 million stroke survivors, with another 9 million new strokes annually (WHO). The majority of stroke victims are not admitted to hospital and diagnosed within 6 h ( Evenson *et al.* , 2009 ) yet clot-busting therapies only work when treatment is initiated well within 4.5 h. New therapies are urgently needed ( Carmichael, 2005 ; Donnan *et al.* , 2008 ).

We are the first to study whether neurotrophin-3 (NT3, encoded by *NTF3* ) can improve recovery when given in a clinically-feasible time frame after stroke. Others have shown that NT3 plays a role in the development, function and repair of locomotor circuits ( Chen *et al.* , 2002 , 2006 , 2008 ; Patel *et al.* , 2003 ; Zhou *et al.* , 2003 ) and reported that intracranial delivery of NT3 immediately following stroke or by intracranial gene therapy prior to stroke reduces infarct volume ( Zhang *et al.* , 1999 *a* , *b* , 2012 ). Moreover, NT3 restores sensorimotor function following spinal cord injury in rats ( Schnell *et al.* , 1994 ; Grill *et al.* , 1997 ; Zhou *et al.* , 2003 ; Fortun *et al.* , 2009 ) by promoting axon growth and synaptic plasticity in multiple locomotor pathways including the corticospinal tract and proprioceptive pathways. All these systems express TRK and/or p75 receptors for NT3 in rodents and primates including humans ( Altar *et al.* , 1993 ; Barrette *et al.* , 2007 ; Brock *et al.* , 2010 ). We therefore examined the ability of NT3 to promote recovery in a model of stroke.

We chose to deliver NT3 by a peripheral route for translational relevance. First, peripheral doses of recombinant NT3 are safe and well tolerated in phase I and II clinical trials for other disorders ( Chaudhry *et al.* , 2000 ; Coulie *et al.* , 2000 ; Parkman *et al.* , 2003 ; Sahenk, 2007 ). Intramuscular injection is a clinically straightforward route after ischaemic stroke in humans. We chose to use a gene therapy system [adeno-associated virus (AAV) serotype 1], which causes effective transgene synthesis in muscles, and is being used clinically in Europe ( Ferreira *et al.* , 2014 ): AAVs cause no identified disease or symptoms in humans and recombinant-deficient AAVs have low immunogenicity or toxicity. NT3 protein is retrogradely transported from muscle to dorsal root ganglion neurons and motor neurons where it causes gene transcription, axon growth of primary afferents, and synapse strengthening within locomotor circuits ( DiStefano *et al.* , 1992 ; Yan *et al.* , 1993 ; Taylor *et al.* , 2001 ; Chen *et al.* , 2002 ; Patel *et al.* , 2003 ; Lee *et al.* , 2012 ; Wang *et al.* , 2012 ). Finally, NT3 may be secreted in the spinal cord after transport in sensory afferents and motor axons ( Zhou and Rush, 1994 ; von Bartheld *et al.* , 1996 ; Wang *et al.* , 2008 ). Thus, delivery of NT3 to the muscle could be a safe and effective way to induce spinal neuroplasticity after stroke. Because >90% of strokes occur in people older than 65 ( Truelsen *et al.* , 2006 ), we evaluated the effectiveness of NT3 in elderly rats as well as adult rats. We chose to deliver our therapy 24 h after cortical ischaemia, because the median time to hospital admission and diagnosis is 6 h in major cities ( Harraf *et al.* , 2002 ; Evenson *et al.* , 2009 ); therefore, this therapy might be applicable to a large number of stroke patients.

We now show that intramuscular injection of AAV1 encoding human NT3 (hNT3) promotes sensory and locomotor recovery in adult rats, even when treatment is initiated 24 h after stroke.

## Materials and methods

### Experimental design

**Figure 1:**
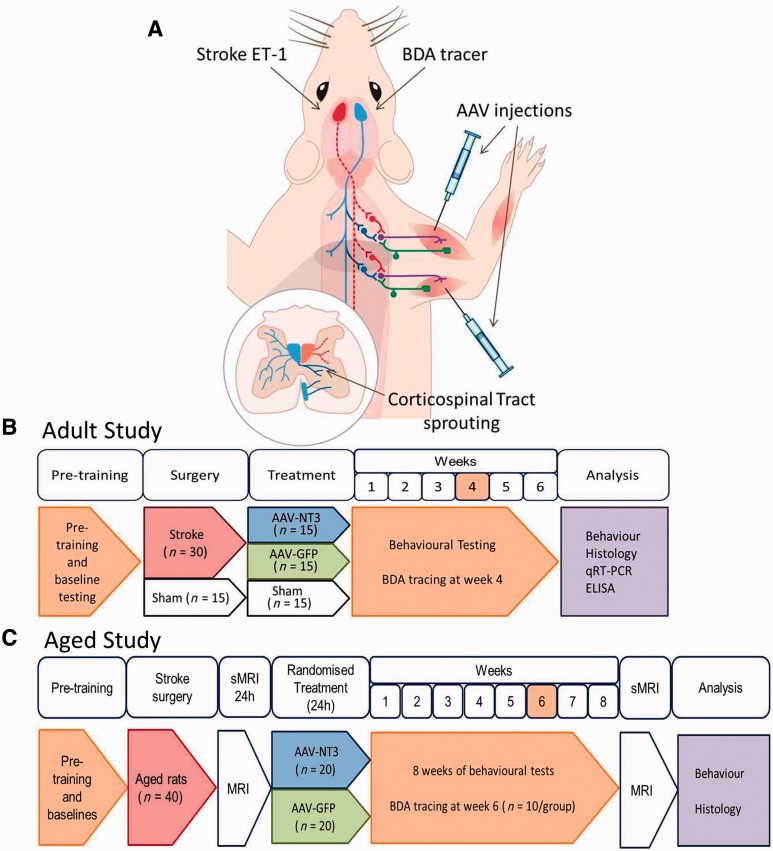
**Design of Experiment 1.** ( **A** ) Schematic, and timelines of the studies using ( **B** ) adult rats and ( **C** ) elderly rats. Rats were pre-trained on the horizontal ladder. Rats then received either stroke or sham surgeries. Stroke was induced by application of endothelin-1 to the sensorimotor cortex ( **A** ; red). One day after stroke either AAV1-hNT3 or AAV1-GFP was injected into the affected forelimb triceps brachii and biceps brachii. Rats underwent ( **B** ) 6 weeks or ( **C** ) 8 weeks of behavioural testing. Anterograde tracer (BDA) was injected into the less affected sensorimotor cortex ( **A** ; blue) 2 weeks prior to the end of each study. Rats were euthanized at the end of the study and tissues were analysed. The experimenter was blinded to all treatments until the end of the study. All surgeries and treatments allocations were randomized.

Forty-five Lister hooded adult female rats were used for the first experiment (6 months old; 200–300 g; outbred) and 40 elderly Long Evans (18 months; 300–600 g; outbred) female rats were used in the second experiment (Charles River). We used Long Evans rats for our second experiment because we had previous experience with inducing stroke in elderly rats of this strain ( Soleman *et al.* , 2012 ), which allowed us to perform appropriate sample size calculations. The experimental designs are presented in Fig. 1 . All surgeries, behavioural testing and analysis were performed using a randomized block design and in a blinded fashion. Allocation concealment was performed by having AAV1-hNT3 and AAV1-GFP stocks coded by a person independent of the study. The blinded treatment code for each rat was drawn at random from a hat without replacement. Codes were only broken after the end of the study. All procedures were in accordance with the UK Home Office guidelines and Animals (Scientific Procedures) Act of 1986. Animals were housed in groups of three to four in Plexiglas® cages with tunnels and bedding, on a 12:12 h light/dark cycle and had access to food and water *ad libitum* .

### Stroke surgery

Animals were anaesthetized with isoflurane (4% in O _2_ for induction) and then transferred to a stereotaxic frame (Kopf). Anaesthesia was maintained at 1.5–2% in O _2_ delivered via a facemask. Rectal temperature was maintained at ∼36°C using a homeothermic system. Ischaemic stroke was induced stereotactically as described previously ( Soleman *et al.* , 2010 , 2012 ) in rats in the cortex representing the dominant forelimb according to preoperative behavioural testing. In stroke rats, a midline incision was made and the sensorimotor cortex was exposed by rectangular craniotomy at the following mediolateral (ML) and anterioposterior (AP) co-ordinates: ML 2 mm to 4 mm, AP 4 mm to −2 mm, relative to Bregma. The dura mater was incised using a 25-gauge needle. For adult rats, three 0.5 µl volumes of endothelin-1 (ET-1, encoded by *Edn1* ) (400 pmol in sterile saline; 0.5 µg/μl; Calbiochem) was administered topically onto the cortical surface (ML 2.8 mm; AP 3.5 mm, 2 mm, −0.5 mm). There was a 2-min interval between each application of ET-1. For aged rats, four 1-μl volumes of ET-1 were administered topically and four 1-µl volumes were microinjected intracortically at the following co-ordinates (relative to Bregma, midline and brain surface, respectively): (i) AP +3.5 mm, ML 2.8 mm, DV −1.0 mm/0 mm; (ii) AP +2 mm, ML 2.8 mm, DV −1.0 mm/0 mm; (iii) AP +0.5 mm, ML 2.8 mm, DV −1.0 mm/0 mm; and (iv) AP −1 mm, ML 2.8 mm, DV −1.0 mm/0 mm.

Prior to suturing, the animal was left undisturbed for 5 min and the skull fragment was replaced. Sham rats received all procedures up to but not including craniotomy as this minor procedure can itself produce behavioural deficits ( Adams *et al.* , 1994 ). Skin was sutured (Vicryl, 4/0, absorbable sutures, Ethicon). Animals were kept in a heated recovery box until fully conscious and analgesia (buprenorphine, 0.01 mg/kg, subcutaneously) was given after suturing and recovery. All of the adult rats and 93% of aged rats survived this surgery.

### Intramuscular injections of AAV1-hNT3

The AAV transfer plasmid, pAAVsp, has a CMV promoter, a synthetic intron flanked by splice donor/splice acceptor sites, and a multiple cloning site terminated by a beta globin polyA sequence. The transcript is flanked by AAV2 inverted terminal repeats. pAAVsp-hNT3 was cloned using the human pre-pro-neurotrophin-3 coding DNA sequence (CDS 8538.1, 774 bp) corresponding to transcript variant 2 (NM_002527.4), which encodes the isoform 2 precursor protein (257 amino acids) including the secretory signal sequence. The hNT3 CDS was restriction digested from a modified pBluescript® plasmid (SKsp) using SfiI and PmeI sites and then cloned into pAAVsp with these same sites. pAAV-EGFP was created by cloning EGFP into the pAAVsp between AgeI and XhoI sites. Plasmids were cloned in the laboratory of Prof. Fred Gage (Salk Institute, CA). AAVs (serotype 1) were generated using the pHelper plasmid and the capsid plasmid encoding Rep/Cap1. Vectors were prepared and titred by PCR (Virapur).

Twenty-four hours after surgery, rats were anaesthetized with isoflurane and a small incision was made between elbow and axilla. Stroke rats received AAV1-GFP or AAV1-hNT3 into triceps brachii and biceps brachii. Injections were spaced at regular intervals and made parallel to the long axis of each muscle using an ultrafine, bevelled non-coring 32-gauge needle (Hamilton) with the intention of targeting the neurovascular bundles and motor end plates that are located in the proximal one-third to one-half of the muscles ( Tosolini and Morris, 2012 ). For triceps, 25 μl was injected deeply into the long head (5 × 5 μl) and the lateral head (5 × 5 μl) and 7.5 μl was injected superficially into the long head (3 × 2.5 μl) and the lateral head (3 × 2.5 μl). For biceps, 15 μl (3 × 5 μl) was injected superficially and 10 µl (2 × 5 μl) deeply. A total of 90 μl was injected into each rat. In total, elderly rats received 7.0 × 10 ^10^ viral genomes (vg) and adult rats received 3.0 × 10 ^10^ vg. Sham rats underwent skin incision without AAV injection. Skin was sutured and analgesic administered as above.

### Behavioural assessment

Rats were trained (for 3 weeks) and evaluated (6 or 8 weeks) on behavioural tasks. Preoperative baseline scores for the horizontal ladder were collected 1 week before surgery.

### Sensory impairments

The bilateral sticky patch test was used ( Schallert *et al.* , 1982 , 2000 ). To identify the affected forelimb, an adhesive label (13 mm diameter, Ryman) was attached to each forelimb wrist surface and the order of label removal was recorded over at least three trials until a 75% preference was observed. A Sensory Impairment Score was determined using seven stimulus pairs ( Fig. 6 A), starting with pair 3; the smaller stimulus was placed on the less affected forelimb and the larger stimulus was placed on the affected forelimb. If the rat removed the stimulus from the less affected limb first, then stimulus size was decreased on the less affected forepaw and increased on the affected forepaw by an equal amount (14.1 mm ^2^ ). This was repeated until the rat finally removed the stimulus on the affected forepaw before the less affected forepaw. The Sensory Impairment Score is derived from the mean of the stimulus pairs used before and after reversal.

### Walking

The apparatus consisted of Plexiglas® side walls, 1.2-m long, 50-cm high and width adjusted to ∼2 cm wider than the animal to try and prevent turning. Metal rungs were placed at a height of 20 cm; they were spaced unequally (between 1 cm and 4 cm apart) and changed weekly to avoid improvement through pattern learning. Rats were videotaped crossing a 1-m length of the horizontal ladder weekly, three times per session. Any slight paw slips, deep paw slips and complete misses were scored as errors. The mean number of errors per step was calculated for each limb for each week. Foot faults are routinely normalized ‘per step’ after stroke ( Metz and Whishaw, 2002 ; Soleman *et al.* , 2010 ): we also checked that there were no differences between groups in the number of steps taken after stroke (linear model *P* -values > 0.05). Moreover, analysis of foot fault data with or without normalization led to the same conclusions being drawn.

### Corticospinal tract tracing

Anterograde tracer [biotinylated dextran amine (BDA) 10 000 kDa, 10% in phosphate-buffered saline (PBS) Invitrogen] was injected into the contralesional sensorimotor cortex 2 weeks before the end of each study [i.e. after 4 weeks in adult rats and after 6 weeks in elderly rats ( Fig. 1 ) after behavioural testing for that day was completed]. Six holes were drilled into the skull using a dental drill at the following coordinates relative to Bregma: (i) AP: +1.0 mm, ML: 1.5 mm; (ii) AP: +0.5 mm, ML: 2.5 mm; (iii) AP: +1.5 mm, ML: 2.5 mm; (iv) AP: +0.5 mm, ML: 3.5 mm; (v) AP: +2.0 mm, ML: 3.5 mm; and (vi) AP: −0.5 mm, ML: 3.5 mm. BDA was injected using a Hamilton syringe and a glass micropipette. The micropipette was slowly lowered 1.5 mm below the cortical surface and BDA injected at a rate of 0.25 μl/10 s with a pause of 1 min after each infusion. The scalp was then sutured and analgesic given as described above. Two weeks after BDA injection, rats were terminally anaesthetized with sodium pentobarbital (Euthatal) and perfused transcardially with PBS (NaCl, 137 mM; KCl, 2.7 mM; Na _2_ HPO _4_ , 4.3 mM; KH _2_ PO _4_ , 1.4 mM) for 2 min, followed by 500 ml of 4% paraformaldehyde (PFA) in PBS for 12 min. The brain, spinal cord and muscles were dissected and stored in 4% PFA in PBS for 2 h, transferred to 30% sucrose in PBS and stored at 4°C. The C1 and C7 spinal cord segment was embedded in gelatin and then post-fixed for 24 h and then cryoprotected in 30% sucrose in PBS. Forty micrometre transverse slices were cut using a freezing stage microtome (Kryomat) and transferred into Tris-buffered saline/azide (100 mM Tris, 15 mM NaCl, 0.5 mM NaN _3_ , pH 7.4) in 24-well plates and stored at 4°C. Ten series of sections were cut and placed in 10 wells.

### Histology

BDA staining was as follows: sections were incubated in 0.3% H _2_ O _2_ and 10% methanol (30 min). Sections were incubated in ABC reagent (VectorLabs) (30 min) then amplified using biotinyl tyramide (1:75, PerkinElmer), then left overnight at 4°C with extra avidin FITC (1:500, Sigma). Sections were washed between steps using PBS.

Series of 40-μm thick transverse sections of fixed spinal cord were immunolabelled as previously described ( Soleman *et al.* , 2010 ). Primary antibodies (overnight) were: rabbit anti-PKCγ (Santa Cruz Biotechnology, 1:500); rabbit anti-CGRP (1:4000, Sigma); rabbit anti-serotonin (1:6000, Sigma). Secondary antibodies (3 h) were: donkey anti-rabbit IgG Alexa 546 (1:1000, Jackson Labs); goat anti-rabbit IgG Alexa 488 (1:1000, Sigma); goat anti-rabbit IgG Alexa 546 (1:1000, Sigma), and DAPI (1:50 000, Sigma). Sections were washed with PBS and then mounted and cover slipped with Mowiol ^®^ .

### Muscle histology and immunolabelling for macrophages

Triceps brachii muscles were dissected from forelimbs of the adult and elderly rats described above (6 and 8 weeks post-injection, respectively). Triceps brachii muscles were also dissected from forelimbs of additional naïve adult (4 month) rats (i.e. no intramuscular injections; *n =* 2) and from adult rats injected with either AAV-NT3 or AAV-GFP at 48 h ( *n =* 2, 2). For a positive control, two naive adult rats were injected with lipopolysaccharide (LPS, 1 mg/ml, Sigma) and sacrificed 48 h later to ensure the antibody staining for macrophages was working optimally. Muscles were cut in transverse sections at 30 µm using a cryostat in a series of five. One series from each rat was stained for monocytes and macrophages, using the pan-macrophage marker CD-68 (mouse anti-CD68, 1:200, Abcam, Ab31630).

Immunofluorescence was visualized under a Zeiss Imager.Z1 microscope or a confocal Zeiss LSM 700 laser scanning microscope. Photographs were taken using the AxioCam and AxioVision LE (Rel. 4.2) and ImageJ was used for image analysis.

Corticospinal axons were counted that crossed the midline, and at two more lateral planes at C7 and at the midline of C1 ( Fig. 4 A). For each rat, the number of corticospinal axons per cord segment were calculated by counting the number of corticospinal axons in all sections in a series and then multiplying by the total number of sections in the whole C7 segment and then divided by the number of sections counted.

### Infarct measurements

**Figure 2:**
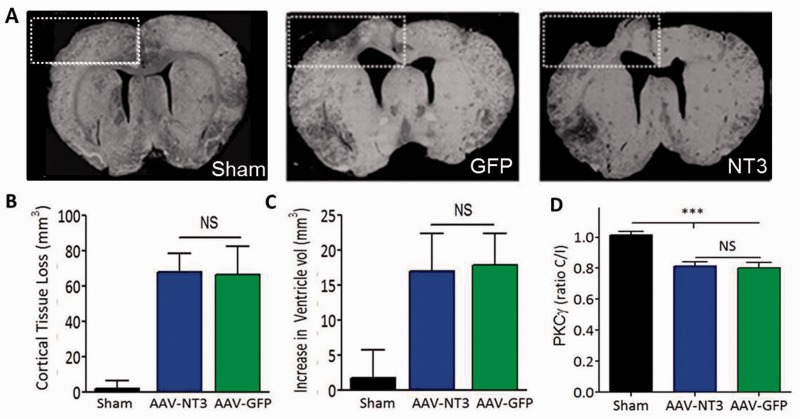
**AAV1-hNT3 does not cause neuroprotection following stroke in adult rats.** ( **A** ) Six weeks after stroke, the infarct volume was assessed by quantifying loss of cortical tissue in a defined region (white dashed box). ( **B** ) There was a significant decrease in cortical tissue on the stroke injury side compared to sham (Kruskal-Wallis *P <* 0.001; Mann Whitney *P* -values < 0.05). There was no difference in the loss of cortical tissue between the AAV1-hNT3 and AAV1-GFP groups (Mann Whitney *P* > 0.05). ( **C** ) Six weeks after stroke there was a significant increase in the volume of the ventricles on the ipsilateral side between the stroke groups and the sham group (Kruskal-Wallis *P <* 0.001; Mann Whitney *P* -values < 0.05); however, there was no difference in volume of the ventricles between the two stroke groups AAV1-hNT3 and AAV1-GFP (Mann Whitney *P* > 0.05). *n =* 10 per group. ( **D** ) The cross-sectional area of the corticospinal tract was assessed using PKCγ immunolabelling. This was decreased at 6 weeks in the stroke groups by similar amounts (AAV1-hNT3, 17.2 ± 2.5%; AAV1-GFP, 17.8 ± 3.1%, *n =* 9/group) compared to the sham rats ( *n =* 8) (Kruskal Wallis, *P* = 0.001; Mann Whitney *P* -values < 0.001; NT3 versus GFP *P* = 0.93). Results displayed as mean ± SEM.

Coronal sections were cut from +3.0 mm to −2.5 mm relative to Bregma. For each section, the total area of each hemisphere or ventricle was obtained using ImageJ contour tracing software. Infarct volume was calculated by subtracting the area of the ipsilesional hemisphere from the area of the contralesional hemisphere within a constant boxed area ( Fig. 2 A) in each section. Ventricular volume was calculated by subtracting the area of the contralesional lateral ventricle from the ipsilesional lateral ventricle in each section. Volume of injury (mm ^3^ ) was calculated as the sum of the area from each section, multiplied by the distance between sections.

### Expression of human and rat mRNA *NTF3*

Six weeks after intramuscular injections in the 6-month-old adult rats, human *NTF3* mRNA (hNT3) was measured in the biceps brachii muscles and ipsilateral C7 spinal hemicords of the rats using quantitative reverse transcriptase polymerase chain reaction (qRT-PCR). Rats were terminally anaesthetized with sodium pentobarbital (Euthatal) and biceps and C7 spinal cord tissues were rapidly dissected and snap frozen in liquid nitrogen prior to storage at −80°C. Total RNA was isolated from biceps brachii and C7 spinal hemicords using TRIzol®. Five hundred nanograms of RNA was DNaseI treated and then cDNA was synthesized using random primers (SuperScript® II, Invitrogen). Quantitative PCR reactions (25 µl) contained 25 ng of cDNA, 25 ng of each primer and 4 µl 5× SYBR® Green PCR Master Mix (Roche). PCR conditions (RotorGene-3000, Corbett Life Science) were: hold (95°C for 10 min), 40 cycles (60°C for 10 s, 72°C for 20 s, 95°C 10 s), extend (72°C 11 s) and melt (72°C to 95°C, 1°C/step, waiting 1 s at the first step and 5 s/step thereafter). Melt curve analyses indicated product specificity. Primers were as follows: hNT3 5’-GAA-ACG-CGA-TGT-AAG-GAA-GC-3’ and 5’-CCA-GCC-CAC-GAG-TTT-ATT-GT-3’, rat NT3 5’-CAG-AAT-TCC-AGC-CGA-TGA-TT-3’ and 5’-CTG-GCC-TGG-CTT-CTT-TAC-AC-3’, GAPDH 5’-ATG-GGA-AGC-TGG-TCA-TCA-AC-3’ and 5’-CCA-CAG-TCT-TCT-GAG-TGG-CA-3’. Standard curves were obtained for each of the target genes (human *NTF3* and rat *Ntf3* ) using 3-fold serial dilutions of foetal human brain cDNA (Stratagene) or embryonic Day 15 (E15) rat head cDNA and melt curve analysis confirmed the specificity of the PCR primers.

### Measurement of NT3 protein

We carried out an additional experiment to explore the biodistribution of NT3 protein using a separate cohort of sham and stroke rats treated with either AAV-hNT3 or AAV-EGFP, and euthanized at 4 days or 8 weeks after stroke ( *n =* 5/group). Endothelin-1 stroke was induced in anaesthetized adult (6 months old) rats on the right sensorimotor cortex (four topical applications, four intracranial injections). Twenty-four hours later left biceps and triceps were injected with AAV-hNT3 or AAV-EGFP. Samples were recovered for ELISA 4 days or 8 weeks later, as well as from sham operated rats. Rats were terminally anaesthetized with sodium pentobarbital (Euthatal). Blood was taken from the heart and allowed to clot overnight at 4°C prior to centrifugation at 14 000 rpm for 15 min on a benchtop centrifuge. The serum was then frozen at −80°C. Other tissues were rapidly dissected and frozen on dry ice prior to storage at −80°C.

After washing with ice-cold PBS, tissue was mechanically homogenized (GentleMACS; Miltenyi Biotech) in RIPA buffer [50 mM Tris HCl pH 7.4, 150 mM NaCl, 1 mM EDTA, 1% Triton ^™^ X-100, 0.1% sodium dodecyl sulphate, containing one tablet of complete mini protease inhibitor cocktail (Roche) per 10 ml] using 10 µl per 1 mg of tissue. Lysates were centrifuged at 13 000 rpm on a benchtop centrifuge, 4°C for 15 min. Supernatants were stored at −20°C. ELISA was performed in duplicates according to the manufacture’s protocol with slight modifications (DuoSet ELISA, R&D Systems). Ninety-six-well plates were coated with the capture antibody at room temperature overnight. Plates were washed three times and blocked for 1 h with Reagent Diluent before 100 µl of sample or blood serum per well was added. Plates were shaken for 5 h at room temperature. After three washes, the secondary antibody was applied overnight, 4°C. The plate was washed, incubated for 20 min with streptavidin-horseradish peroxidase, and developed with substrate solution for 20 min before adding stop solution. The plate was read at 450 nm and 540 nm (SpectraMax 340PC; Molecular Devices). After subtracting the 540 nm readings from the 450 nm readings, NT3 concentration was calculated based on a linear standard curve ranging from 600 pg/ml to 0 pg/ml. Values were normalized to total protein concentration, measured with the Bicinchoninic Acid Assay (Novagen).

### Magnetic resonance imaging

Structural MRI was conducted 1 day following stroke before the treatment was initiated and then at the 8 week end of study time point using a 7 T horizontal bore VMRIS scanner (Varian). Animals were anaesthetized using 2% isoflurane, in 0.8 l/min medical air and 0.2 l/min medical O _2_ in an induction chamber. Once anaesthetized they were secured in a stereotaxic head frame inside the quadrature birdcage magnetic resonance coil (43 mm internal diameter) and placed into the scanner. Rectal temperature was maintained at 37 ± 1°C. Physiology was monitored using pulse oximetry (Nonin) and a respiration monitor (BIOPAC). The T _2_ -weighted MRIs were acquired using a fast spin-echo sequence: effective echo time 60 ms, repetition time 4000 ms, field of view 40 × 40 mm, acquisition matrix 128 × 128, acquiring 20 × 1-mm thick slices in ∼8 min. Data were analysed using a semi-automatic contour method in Jim software (Xinapse). MRI data were not provided or analysed until the end of the study (D.C.) and did not influence randomization.

### Functional magnetic resonance imaging

Alpha chloralose anaesthesia was prepared by mixing equal amounts of borax decahydrate and alpha chloralose (Sigma, UK; <20% beta isoform, catalogue number C0128) in physiological saline to a concentration of 50 mg/ml. The mix was dissolved in a glass beaker under 52°C and then was filtered using a 0.22 μm filter. Animals were anaesthetized using 4–5% isoflurane for induction and 2.5% for maintenance, in 0.8 l/min medical air and 0.2 l/min medical O _2_ in an induction chamber. Tail cannulation was performed and the animal was transferred to the MRI scanner. A bolus of 65 mg/kg alpha chloralose was injected intravenously and then the isoflurane was switched off after 5 min. An infusion line for continuous application of alpha chloralose was then attached to the cannula which provided 30 mg/kg/h over the experimental time. Medical air (0.8 l/min) and oxygen (0.2 l/min) were continuously delivered throughout the scanning period.

Functional MRI was performed in a subset of elderly rats ( *n =* 10/group) at 8 weeks after stroke. Blood oxygen level-dependent functional MRIs were acquired during stimulation of the affected or less affected wrist at non-noxious mechanoreceptor intensity using methods previously established by our neuroimaging team ( Lowe *et al.* , 2007 ). These rats did not receive intracortical injections of BDA tracer. Functional MRI scans were acquired using a 7 T scanner as above and a gradient echo-multi-echo sequence with repetition time = 360 ms, echo times = 5, 10, 15 ms, voxel size 0.5 mm × 0.5 mm × 1 mm, resolution 64 × 64 × 20, scan time 23 s, mean echo time images were analysed. One block of 100 volumes were acquired per wrist with a pseudorandom on-off stimulation of the wrist at 3 Hz (400 μs, 2 mA pulse) using a platinum subdermal needle electrode and a TENS (transcutaneous electrical nerve stimulation) pad. The order of wrist stimulation was also randomized. Rats were terminally anaesthetized using Euthatal and perfused for histology as described above.

Scans with obvious imaging artefacts were discarded, leaving final group numbers of *n =* 5 in the aged AAV-GFP group and *n =* 7 in aged AAV-NT3. Individual masks for each rat brain at each time-point were generated from a fast spin echo (FSE) structural scan using a 3D Pulse-Coupled Neural Network ( Chou *et al.* , 2011 ). The resulting images were analysed with SPM-8 (Statistical Parametric Mapping, FIL, UCL). The scans were mirrored about the sagittal mid-plane, if necessary, so that the lesioned hemisphere always appeared on the left. The first volume of the functional scan was spatially registered to the structural image, which was, in turn, linearly warped to a template brain. Linear warping was used in this step in order to avoid deforming the lesion region. After movement correction of the functional time-series, warping parameters obtained during registration of structural image to template were applied to the functional time-series, resulting in structural and functional images that are all in a standard space. Finally, functional images were smoothed using a Gaussian kernel with full-width at half-maximum of 1.25 × 1.25 × 2 mm. Because of the relatively long effective repetition time of the functional images, a PET basic model (one-sample *t* -test) was used for first-level analyses with covariates consisting of the pseudo-random stimulation pattern, and the estimated movement parameters of each individual rat. The mask created from the structural image (also registered to the template) was used as an explicit mask for the first-level statistical analysis and a global scaling was applied. Contrast images from the first-level analysis were then carried onto a second-level (random effects) group analysis. Effects of group (i.e. NT3- or GFP-treated) and stimulated wrist (i.e. affected or less-affected) were used to create statistical comparisons. This looked to compare the group average response of AAV-NT3 and AAV-GFP to each other during stimulation of the affected or less-affected wrist.

### Statistical analysis

Graphs show means ± standard error of the mean (SEM) and ‘ *n* ’ denotes number of rats. Threshold for significance was 0.05. Two-sided tests were used throughout although one-sided tests were used for ELISAs, given that we predicted an increase in NT3 with NT3 treatment. Asterisks indicate * *P* ≤ 0.05, ** *P* ≤ 0.01 and *** *P* ≤ 0.001. Histology, MRI lesion volumes and molecular biology data were assessed using the non-parametric Kruskal-Wallis and Mann-Whitney tests. Behavioural data from the first study were analysed using repeated measures analysis of covariance and Bonferroni *t* -tests for group differences as the study was balanced and there were no missing values. Behavioural data from the second study were analysed using linear models and Restricted Maximum Likelihood estimation to accommodate data from elderly rats with occasional missing values ( Gueorguieva and Krystal, 2004 ; Krueger and Tian, 2004 ). Akaike’s Information Criterion showed that the model with best fit for the horizontal ladder data had a compound symmetric covariance matrix, whereas for the sensory test data an unstructured covariance matrix was used ( Heck *et al.* , 2010 ). Where measured, baseline scores were used as covariate. Normality was checked using histograms. Homogeneity of variances was checked using Levene’s test. If sphericity was violated (Mauchly’s test) the Greenhouse-Geisser correction was applied. Degrees of freedom are reported to the nearest integer. Sample size calculations were presented previously ( Soleman *et al.* , 2010 ). SPSS (version 22) was used.

## Results

Ischaemic stroke was induced in the sensorimotor cortex representing each rat’s dominant forearm ( Fig. 1 ), using standard methods to induce focal vasoconstriction using endothelin-1 ( Soleman *et al.* , 2010 , 2012 ). Twenty-four hours later, rats were randomized to treatment with treatment allocations concealed using coded vials. Disabled biceps brachii and triceps brachii were injected with either AAV1 encoding human NT3 or AAV1 encoding green fluorescent protein (GFP) ( Fig. 1 ). We strove to inject the belly of these muscles where the neurovascular bundles containing sensory afferents, motor axons and blood supply is found ( Tosolini and Morris, 2012 ). Recovery of sensory and locomotor performance was assessed weekly for 6 weeks in adult rats and 8 weeks in elderly rats. Anterograde tracer was injected into the less-affected sensorimotor cortex 2 weeks before the end of each study. All surgeries and treatments were performed using a randomized block design and the experimenter was fully blinded to treatment allocation until all analyses were completed. The rats were euthanized at the end for either histology or molecular biology.

In the first study, 6 weeks after stroke, histology showed focal infarcts involving motor cortex and somatosensory cortex of both the forelimb and hindlimb regions ( Fig. 2 A). There were no differences between stroke groups in lesion volumes ( Fig. 2 B). The ventricles were increased in volume on the affected side ( Fig. 2 C) and there were no differences between stroke groups. The two stroke groups also showed a comparable loss of corticospinal tract axons as assessed in the upper cervical dorsal columns using protein kinase C gamma (PKCγ) immunofluorescence ( Fig. 2 D). Therefore, as expected from our previous work, stroke causes a focal infarct in sensorimotor cortex and loss of cortical efferents to the cervical spinal cord, and delayed treatment with NT3 does not cause neuroprotection (consistent with the delayed time frame of administration).

### AAV1-hNT3 treatment improved arm locomotor function in rats after stroke

**Figure 3:**
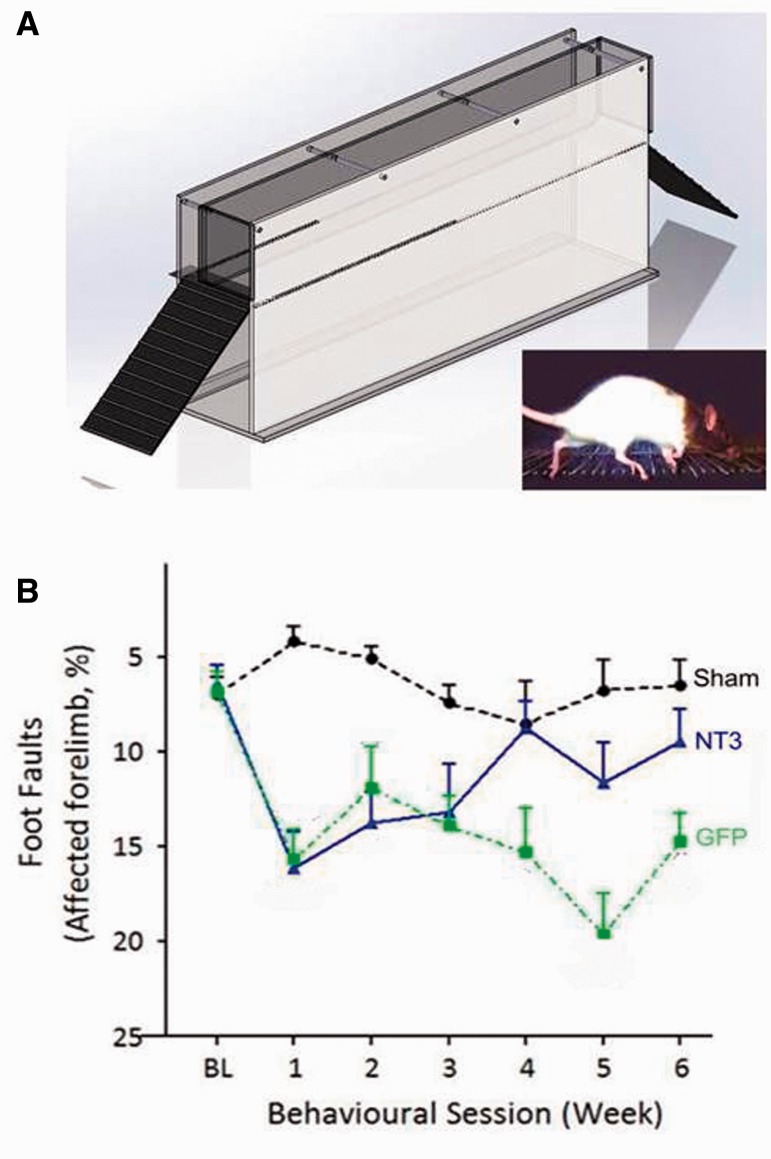
**Following stroke, delayed AAV1-hNT3 improves walking on a horizontal ladder with irregularly spaced rungs.** ( **A** ) A horizontal ladder with irregularly spaced rungs was used to evaluate locomotion. ( **B** ) One week after stroke, these two groups made a similar number of errors with their affected forelimb ( *t* -test *P =* 0.78). The AAV1-hNT3 group recovered compared to the AAV1-GFP group [group *F* (2,41) = 29.7, *P* < 0.001; *post hoc P =* 0.024]. By Week 4 the AAV1-hNT3 group made no more errors than shams, whereas the AAV1-GFP group remained impaired relative to shams at Week 6 [group × time *F* (7,149) = 2.7, *P =* 0.011; *post hoc P* -values = 0.93 and < 0.001, respectively]. Repeated measures ANCOVA and *post hoc* Bonferroni tests. Mean ± SEM. *n =* 15/group.

The horizontal ladder task was used to measure forelimb recovery following stroke injury ( Fig. 3 A). The rungs were spaced at 1–4-cm intervals and changed weekly to avoid pattern learning. This task evaluates sensory-guided paw placements and corticospinal-dependent motor control ( Metz and Whishaw, 2002 ). One week after stroke, these two groups made a similar number of errors with their affected forelimbs when crossing a horizontal ladder ( Fig. 3 B). The AAV1-hNT3 group progressively recovered compared to the AAV1-GFP group. Thus, delayed intramuscular treatment with AAV1-hNT3 improved locomotor function after ischaemic stroke in rats. Rats showed some session-to-session variability in performance. For example, both stroke groups transiently declined at Week 5 on the ladder test: this might be due to injection of anterograde tracer into the less affected cortex (6 days previously).

### Treatment promoted sprouting of cortical efferents into the affected spinal hemicord

Anterograde tracer (biotinylated dextran amine, BDA) was injected into the less affected sensorimotor cortex 2 weeks before the end of the study ( Fig. 1 ). Immunolabelling for BDA was used to assess axonal sprouting of the intact corticospinal tract into the partially denervated side of the spinal cord grey matter following AAV1-hNT3 treatment. We chose to evaluate the corticospinal tract in cervical segments 7 and 1 because the triceps brachii and biceps brachii are supplied by dorsal root ganglia and motor neurons between C3 and T1 ( McKenna *et al.* , 2000 ; Tosolini and Morris, 2012 ); accordingly, we hypothesized that NT3 would either directly or indirectly induce corticospinal sprouting at C7 but not C1.

**Figure 4:**
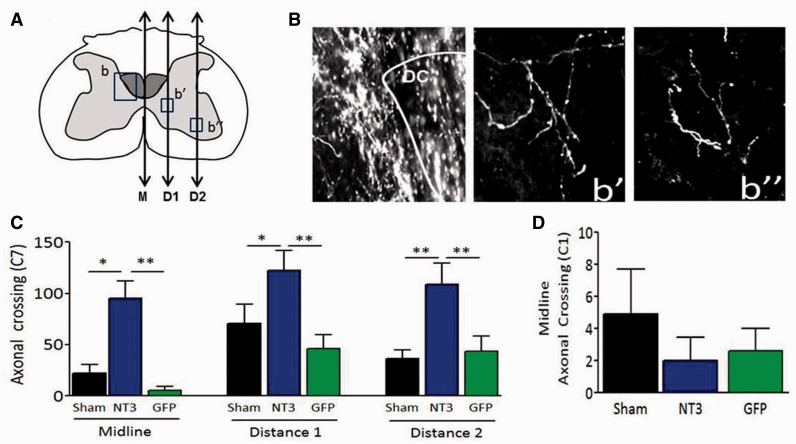
**Delayed AAV1-hNT3 increases sprouting of spared corticospinal axons in the spinal cord.** ( **A** ) Schematic of C7 spinal cord showing the planes (ipsilateral to treatment) at which axonal crossing was measured, defined as Midline (M), Distance 1 (D1) and Distance 2 (D2). ( **B** ) Photomicrographs of corticospinal axons from an AAV1-hNT3 treated rat in the (b) dorsal columns, and at (b’) Distance 1 and (b’’) Distance 2. ( **C** ) Increased corticospinal sprouting in AAV1-hNT3 rats at C7 at M, D1 and D2 (Kruskal-Wallis *P* -values ≤ 0.001, 0.02 and 0.023, respectively; Mann-Whitney *P* -values all < 0.011; *n =* 7 to 10/group). ( **D** ) There was no difference in corticospinal crossing at C1 midline (Kruskal-Wallis *P =* 0.73, *n =* 8 to 10/group). Mean ± SEM.

Corticospinal axonal sprouting was measured at three parasagittal planes of the spinal cord at cervical levels C7 and C1 ( Fig. 4 A). Figure 4 B shows representative pictures of corticospinal axons in the dorsal columns and at the two lateral planes. Statistical analysis revealed that there was a significant difference between the three groups at C7 ( Fig. 4 C) with significantly more corticospinal collateral sprouting in the AAV1-hNT3 treated group at the midline and two more lateral planes compared to the sham and control AAV1-GFP groups. As predicted, this increase in sprouting was not evident in the cervical C1 segment ( Fig. 4 D). This shows a level-specific effect of NT3 on corticospinal axonal sprouting.

### Human *NTF3* mRNA was synthesized in affected biceps and triceps but not in the spinal cord

**Figure 5:**
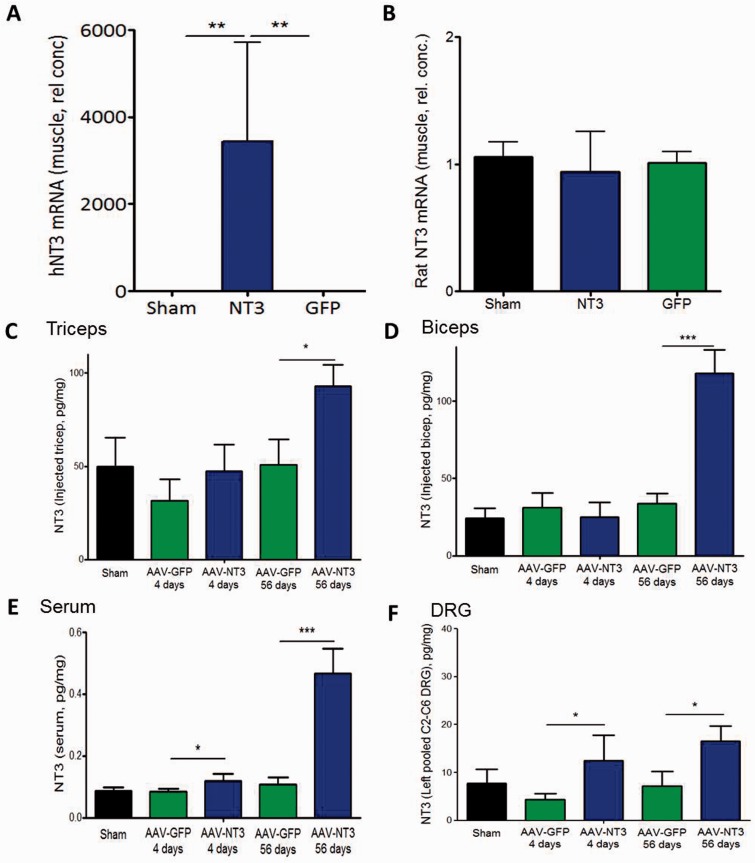
**Intramuscular AAV1-hNT3 caused an elevation of NT3 protein in injected muscles, serum, and ipsilateral cervical dorsal root ganglion cells.** ( **A** ) Quantitative reverse transcriptase PCR showed high levels of human *NTF3* mRNA (hNT3) in injected biceps brachii compared to AAV1-GFP rats or shams (Kruskal-Wallis, *P =* 0.009; Mann-Whitney *P* = 0.008 and 0.008, respectively, *n =* 5/group). ( **B** ) There was no difference between treatment groups in the level of endogenous rat *Ntf3* mRNA (Rat NT3) level in the injected muscle (Kruskal-Wallis *P* -value > 0.05, *n =* 5/group). ( **C** ) NT3 protein was significantly elevated in treated triceps brachii in stroke rats at 8 weeks after AAV1-hNT3 administration ( *P =* 0.018) but not detectably at 4 days ( *P =* 0.21). ( **D** ) NT3 was elevated in treated biceps brachii in stroke rats at 8 weeks after AAV-hNT3 administration ( *P* < 0.001) but not detectably at 4 days ( *P =* 0.33). ( **E** ) NT3 increased in the serum at 4 days ( *P =* 0.039) and 8 weeks after AAV1-hNT3 administration ( *P* < 0.001). ( **F** ) NT3 was increased in C2–C6 dorsal root ganglia on the treated side in stroke rats at 4 days ( *P =* 0.019) and 8 weeks after administration of AAV1-NT3 ( *P =* 0.031). Mean ± SEM, *n =* 5/group, *t* -test versus time-matched GFP control; * *P* < 0.05, ** *P* < 0.01, *** *P <* 0.001.

Six weeks after intramuscular injection, human *NTF3* mRNA was measured in the biceps brachii of five randomly selected rats per group using quantitative reverse transcriptase PCR. Although the human and rat mature NT3 proteins are identical, the mRNAs for human and rat NT3 differ and can be distinguished using quantitative reverse transcriptase PCR. As expected, rats injected with AAV1-hNT3 had high levels of human *NTF3* mRNA in their biceps brachii muscles ( Fig. 5 A) whereas this was undetectable in rats injected with AAV-GFP or in shams. We also measured the level of endogenous rat *Ntf3* mRNA in the muscle to see whether intramuscular AAV1-hNT3 had boosted host synthesis of *Ntf3* mRNA. There was no significant difference in the level of endogenous rat *Ntf3* mRNA in the injected muscle of the AAV1-hNT3 treated rats compared to sham or AAV1-GFP treated rats ( Fig. 5 B). Thus, as expected, human *NTF3* mRNA was highly expressed in the injected muscle of AAV1-hNT3 treated rats.

### NT3 protein was elevated in ipsilateral cervical dorsal root ganglion and in serum

We explored the biodistribution of NT3 protein using a second cohort of sham and stroke rats treated with either AAV1-hNT3 or AAV1-EGFP, and killed at 4 days or 8 weeks after stroke. Stroke was induced in anaesthetized adult rats in the right sensorimotor cortex. Twenty-four hours later, the left biceps brachii and triceps brachii were injected with AAV1-hNT3 or AAV1-EGFP. Samples were recovered for quantitative reverse transcriptase PCR and ELISA 4 days or 8 weeks later, as well as from sham operated rats.

In treated triceps ( Fig. 5 C), NT3 protein was present in sham rats and in stroke rats at 4 days and 8 weeks after AAV-EGFP administration. NT3 protein was significantly elevated in treated triceps in stroke rats at 8 weeks after AAV1-hNT3 administration. In treated biceps ( Fig. 5 D), modest levels of NT3 protein were detectable in sham rats and in stroke rats at 4 days and 8 weeks after AAV-EGFP administration. NT3 protein was elevated in treated biceps in stroke rats at 8 weeks after AAV1-hNT3 administration. In serum ( Fig. 5 E), basal levels of NT3 protein were low but detectable in sham rats and in stroke rats at 4 days and 8 weeks after AAV1-EGFP administration. NT3 protein was elevated in serum in stroke rats at 4 days and 8 weeks after AAV1-hNT3 administration. ELISA of pooled homogenates of left C2 to C6 dorsal root ganglion ( Fig. 5 F) showed that NT3 was increased on the treated side in stroke rats at 4 days and 8 weeks after administration of AAV1-NT3 into triceps and biceps. In conclusion, our experiments show that NT3 was synthesized in the biceps brachii and triceps brachii and then transported to ipsilateral cervical dorsal root ganglions as well as being secreted into serum.

### NT3 improved sensory and motor function in elderly rats

**Figure 6:**
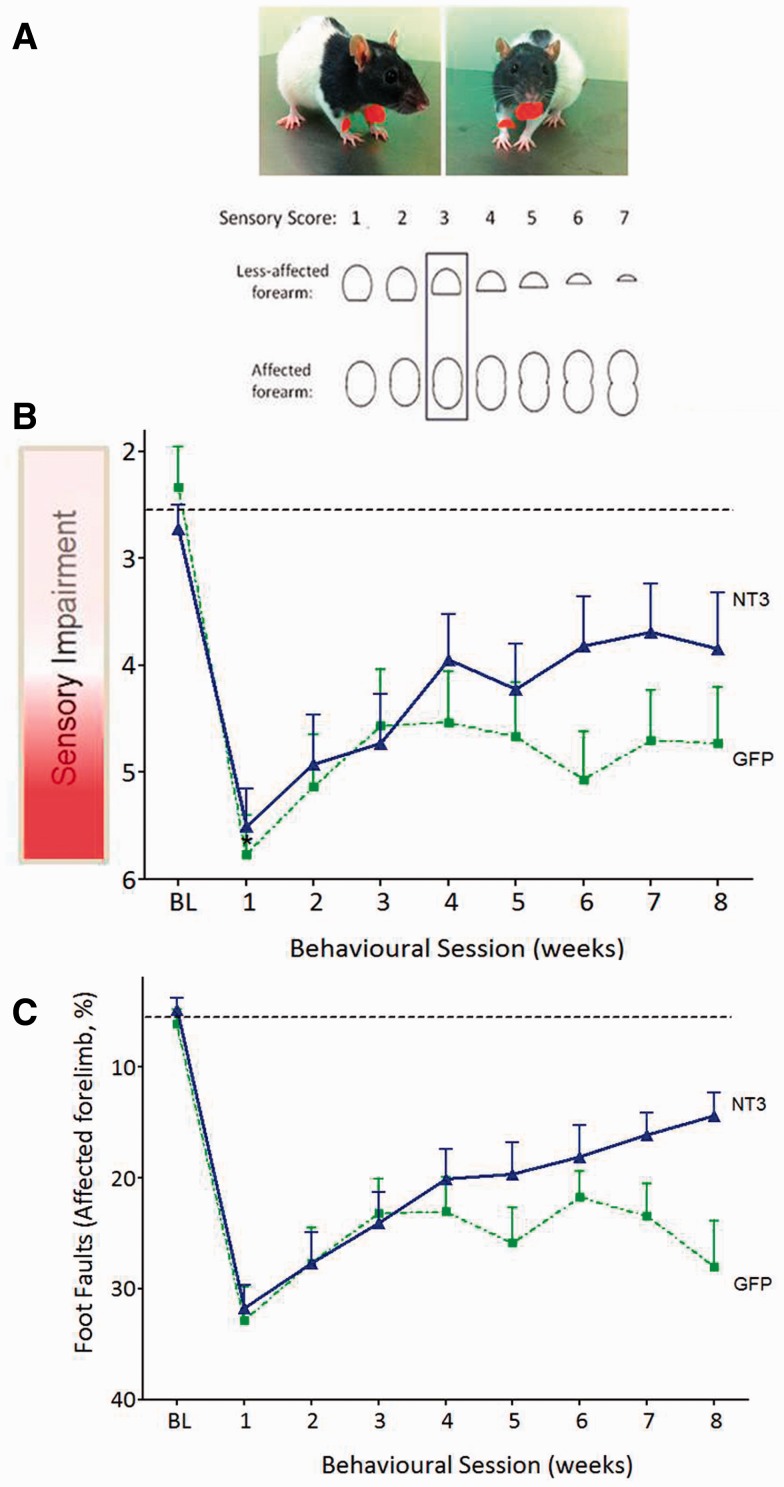
**
Delayed treatment of disabled arm muscles with AAV1 expressing human *NTF3* reversed neglect and improved walking after stroke in elderly rats.** ( **A** ) Schematic of the seven pairs of stimuli that were used to evaluate tactile impairment. Each test started at level 3 (boxed). ( **B** ) A photograph showing the position that the sticky labels were adhered to on each rat’s forelimbs. ( **C** ) After 1 week, the two groups of stroke-injured rats had similar, large sensory scores ( *P =* 0.48). Treatment with AAV1-hNT3 caused a progressive recovery from this neglect relative to controls [treatment *F* (1,30) = 5.2, *P =* 0.030]. ( **D** ) One week after stroke, the two groups made a similar number of errors with their affected forelimbs ( *t* -test *P =* 0.50). Treatment with AAV1-hNT3 caused a progressive recovery of their affected forelimbs relative to controls [interaction of treatment × time *F* (7,225) = 2.15, *P =* 0.040].

Next, we assessed whether AAV1-hNT3 could improve recovery in elderly rats when treatment was initiated 24 h after stroke ( Fig. 1 C). We extended the length of the study to see whether more recovery would be obtained. We used the bilateral ‘adhesive patches’ test to assess tactile extinction (hereafter ‘neglect’) ( Schallert *et al.* , 1982 ; Schallert and Whishaw, 1984 ), which is a phenomenon manifested in many stroke patients who may fail to detect a touch stimulus on their affected hand if stimuli are applied simultaneously on both hands ( Doyle *et al.* , 2010 ). This test involves systematic application of seven pairs of adhesive labels to the rat’s wrists ( Fig. 6 A and B). A Sensory Impairment score of 6 denoted that a rat neglected a very large stimulus on its affected paw and preferentially removed the smaller stimulus from its less affected paw. After 1 week, the two groups of stroke-injured elderly rats had similar, large Sensory Impairment scores indicating that both groups neglected the larger stimulus on their affected forepaw and preferentially removed the smaller stimulus from their less affected forepaw. Treatment with AAV1-hNT3 caused a progressive recovery from this neglect relative to controls ( Fig. 6 C). Walking on the horizontal ladder was assessed as before. One week after stroke, the two groups made a similar number of errors with their affected forelimb. Treatment with AAV1-hNT3 caused a progressive recovery of their affected forelimb relative to controls ( Fig. 6 D).

**Figure 7:**
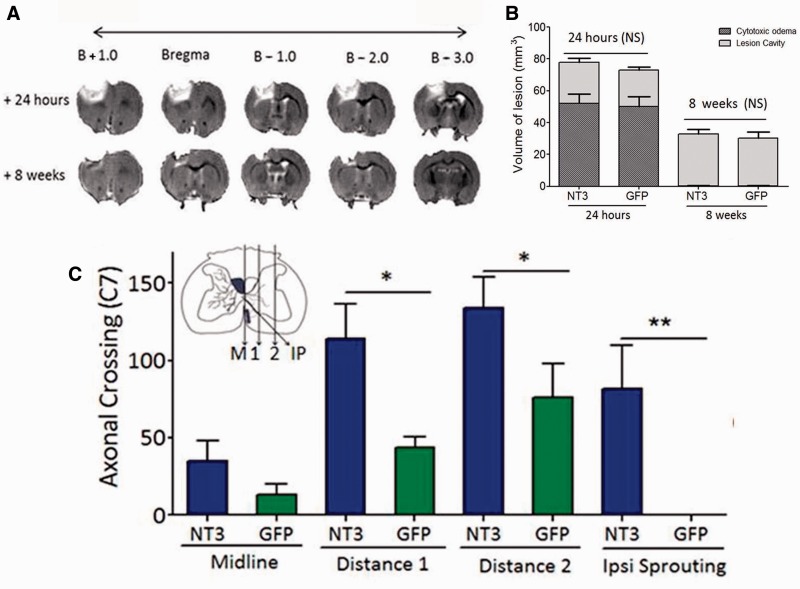
**In elderly rats, recovery was not due to neuroprotection; instead, delayed treatment of disabled arm muscles with AAV1-hNT3 induced neuroplasticity in corticospinal axons.** ( **A** ) T _2_ -weighted MRIs from the same rat at 24 h and at 8 weeks after stroke showing oedema at 24 h, which resolves by 8 weeks leaving a lesion core. ( **B** ) MRI confirmed there were no differences between groups in infarct size either at 24 h or 8 weeks after stroke (Mann Whitney tests *P* = 0.56 and 0.23, respectively). Light bars represent lesion core whereas dark bars represent oedema. ( **C** ) *Inset* shows planes (ipsilateral to treatment) where corticospinal axons were counted. AAV1-hNT3 caused increased corticospinal sprouting at C7 at distances 1 and 2 and from the ipsilateral (IP) ventral corticospinal but not at the midline (Mann-Whitney *P* -values = 0.027, 0.043, 0.010 and 0.32, respectively; *n =* 8–10/group).

MRI showed that 24 h after stroke, infarcts consisted of lesion plus surrounding oedema whereas by 8 weeks the oedema had resolved from the lesion surround ( Fig. 7 A). Infarct volumes did not differ between groups either 24 h after stroke (immediately prior to treatment) or at 8 weeks ( Fig. 7 A and B). Stroke caused a unilateral loss of ∼20% of corticospinal axons with no differences between groups, assessed in the upper cervical dorsal columns using PKCγ immunofluorescent labelling (data not shown). These data show that delayed AAV1-hNT3 treatment did not neuroprotect the elderly brain, as expected. Anterograde tracing showed that corticospinal axons from the less affected hemisphere sprouted in the C7 cervical spinal cord ipsilateral to the AAV1-hNT3 injections ( Figs 1 and 7 C).

To see whether any recovery of somatosensory cortex activation might explain the modest somatosensory recovery (shown in sticky patch testing) after AAV-NT3 treatment, we performed functional brain imaging (blood oxygen level-dependent functional MRI) during stimulation of the affected wrist 8 weeks after stroke ( Lowe *et al.* , 2007 ). We found no differences between groups in perilesional reactivation ( Supplementary Fig. 1 ). Indeed, after correction for testing of multiple voxels, there was no evidence for changes in any of the brain’s voxels after AAV-NT3 treatment relative to AAV-GFP during stimulation of the affected wrist or the less affected wrist (data not shown as heat maps were black). We discuss these results below in more detail.

### Intramuscular injections evoked only a transient, minor inflammatory response

**Figure 8:**
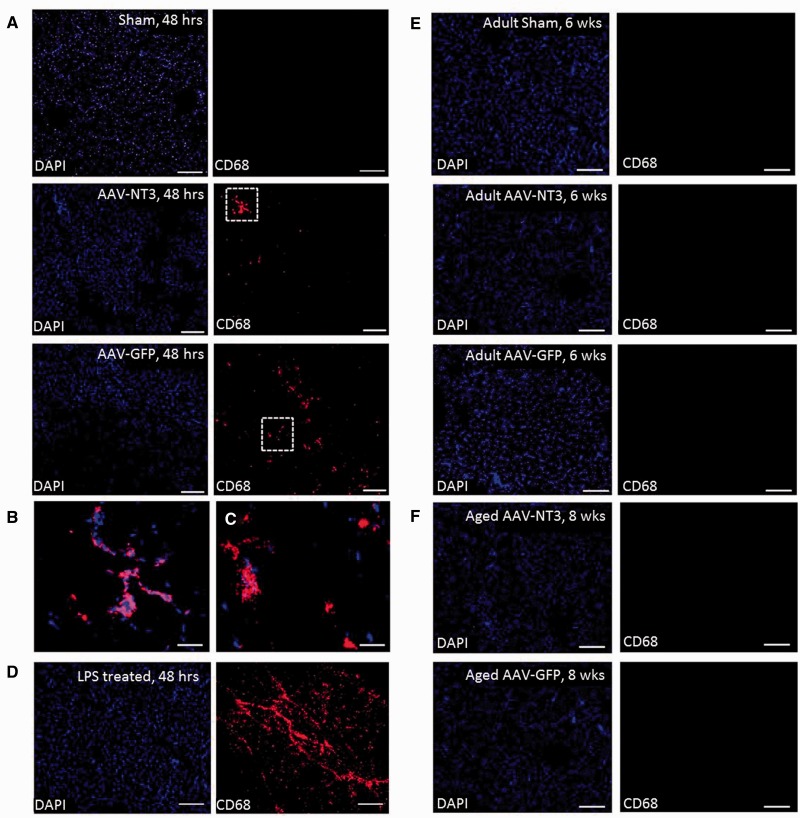
**Intramuscular AAV injections caused only a transient inflammatory response that resolved completely with time.** ( **A** ) Sham adult rats were given no intramuscular injections whereas other adult rats were given injections of AAV-NT3 or AAV-GFP unilaterally into triceps brachii. Forty-eight hours later, immunolabelling for a pan-macrophage and monocyte marker (CD68) showed no cells of the macrophage or monocyte lineage in triceps of sham rats whereas AAV-NT3 and AAV-GFP injected rats had a modest infiltrate. ( **B** and **C** ) Regions in dotted boxes are shown at higher magnification. ( **D** ) As a positive control, other adult rats were injected with LPS. Forty-eight hours later, CD68 immunolabelling showed strong, widespread influx of inflammatory cells. ( **E** ) Few or no CD68 positive inflammatory cells were detected in triceps of sham adult rats or adult rats injected 6 weeks previously with AAV-NT3 or AAV-GFP. ( **F** ) Few or no CD68 positive inflammatory cells were detected in triceps of elderly rats injected 8 weeks previously with AAV-NT3 or AAV-GFP. Red shows CD68; blue shows DAPI. Scale bars: **A** = 200 µm, **B** and **C** = 50 µm; **D** and **E** = 200 µm.

Rats exhibited some session-to-session variability in performance that might indicate deterioration (e.g *.* due to inflammation in the muscle evoked by injection). To explore whether there was any ongoing inflammation in the muscle evoked by AAV injection, we immunolabelled muscles for CD68, which is a pan-macrophage and monocyte marker. As expected, sham adult rats that had no intramuscular injection exhibited few if any macrophages or monocytes ( Fig. 8 A). Adult rats that had injections of AAV-NT3 or AAV-GFP unilaterally into triceps brachii had a modest infiltration of macrophages/monocytes at 48 h ( Fig. 8 A–C). As a positive control, adult rats that had injections of lipopolysaccharide unilaterally into triceps brachii had a massive infiltration of macrophages/monocytes at 48 h ( Fig. 8 D). In contrast, 6 weeks after injection of AAV-NT3 or AAV-GFP into adult rats, few or no inflammatory cells could be detected in the muscles ( Fig. 8 E). Immunolabelling of triceps of elderly rats revealed few or no inflammatory cells 8 weeks after intramuscular injection of AAV-NT3 or AAV-GFP ( Fig. 8 F). Thus there was no ongoing inflammation in either AAV group that could explain any apparent deterioration in the AAV-GFP group.

We suggest that the session-to-session variability reflects performance variability of rodents with small to medium sized strokes (rather than deterioration), as has been seen by other investigators [ Figs 3 and 4 in Tennant *et al.* (2015) ]. In ongoing work we have improved consistency in precision of estimates of performance by using larger numbers of rats per group. Importantly, we have shown that NT3 improves recovery of function in two additional studies with no evidence for any decline of function in the control groups ( Duricki *et al.* , in preparation) so we predict that the benefits of NT3 that we describe in this current manuscript will be reproducible by others.

In summary, the data from these studies showed, consistently, that adult and elderly rats recover some sensory and locomotor function after stroke when treatment using NT3 is initiated starting 24 h after stroke.

## Discussion

These studies show that 24 h delayed intramuscular injection of an AAV1 encoding human NT3 improves sensorimotor recovery in adult and elderly rats after focal cortical stroke. Human *NTF3* mRNA was abundant in injected muscles and NT3 protein was elevated in injected muscles, in serum and in ipsilateral cervical dorsal root ganglion. In both studies, NT3 induced neuroplasticity in the less affected corticospinal tract.

### NT3 induced sensorimotor recovery via neuroplasticity and not neuroprotection

MRI and histology showed that lesion volumes were similar between stroke groups, ventricular expansion was similar between stroke groups, and the extent of corticospinal loss was similar between stroke groups. This lack of neuroprotection is consistent with the fact that AAV1-hNT3 was not administered until 24 h after onset of stroke (i.e. most cell death has already occurred).

Anterograde tracer was injected into the intact (contralesional) sensorimotor cortex to label the spared corticospinal tract. Treatment with AAV1-hNT3 resulted in increased corticospinal axonal sprouting from the intact side to the affected side across the midline and at two further lateral planes. This neuroplasticity of corticospinal fibres was seen in the cervical C7 region, which is innervated by sensory and motor axons that innervate the treated muscle ( McKenna *et al.* , 2000 ; Tosolini and Morris, 2012 ). We and others have shown that NT3 is retrogradely transported from muscles in sensory afferents and motor axons ( DiStefano *et al.* , 1992 ; Zhou and Rush, 1994 ; Curtis *et al.* , 1998 ; Petruska *et al.* , 2007 , 2010 ; Wang *et al.* , 2008 ) and this is consistent with their expression of receptors for NT3 including trkC ( McMahon *et al.* , 1994 ; Sheard *et al.* , 2002 ).

We are continuing to evaluate how NT3 enhances recovery of cutaneous sensory function (i.e. responsiveness to sticky patches on the wrists). Here, we performed functional brain imaging during stimulation of the affected wrist in elderly rats 8 weeks after AAV-NT3 or AAV-GFP. We found no differences between groups in blood oxygen level-dependent functional MRI maps. This is also consistent with another study from our laboratory ( Duricki *et al.* , in preparation). Accordingly, we now hypothesize that changes outside of the brain are responsible for the recovery of cutaneous sensation. In other work, we are now testing the hypothesis that NT3 enhances recovery of sensory function by causing corticospinal axons to increase connectivity with dorsal horn interneurons on the affected side, where corticospinal axons are known to modulate cutaneous and proprioceptive afferent input to the cord ( Seki *et al.* , 2003 ; Abraira and Ginty, 2013 ; Levine *et al.* , 2014 ; Bourane *et al.* , 2015 ). In other work from our lab ( Duricki *et al.* , in preparation), NT3 has been found to enhance sprouting of corticospinal axons into the dorsal horn (laminae I to IV) on the affected side including amongst PKCγ positive interneurons in lamina II. These neurons receive cutaneous inputs from hairy skin, which enables mammals to ‘detect the presence of foreign objects on their skin’ ( Abraira and Ginty, 2013 ), among other things. PKCγ-positive interneurons normally receive corticospinal input, so this plasticity is plausible. Moreover, cutaneous afferent activity can be modified by muscle afferent fibre activity ( Seki *et al.* , 2003 ). Specifically, cutaneous input to the CNS can be inhibited presynaptically by muscle spindle and tendon organ afferents as well as by supraspinal pathways. Thus NT3 might modify cutaneous input via muscle afferent activity and corticospinal activity at the level of the spinal cord.

NT3 is distributed from muscle via axonal transport and the bloodstream. The vasculature at the dorsal root ganglion is highly fenestrated and molecules as large as NT3 can enter and bind ( Jimenez-Andrade *et al.* , 2008 ). We show that NT3 accumulates in the dorsal root ganglion and others have shown that it can enter the brain and spinal cord ( Poduslo and Curran, 1996 ; Pan *et al.* , 1998 ). Accordingly NT3 might bind its receptors that are expressed by many neurons in the sensory pathway from periphery to brain, including cutaneous and proprioceptive afferents ( McMahon *et al.* , 1994 ), dorsal column projections to the brainstem nuclei, and neurons within the thalamus and somatosensory cortices. In ongoing work we are continuing to explore how NT3 modifies spinal cord processing of cutaneous and proprioceptive input.

In future experiments we will determine whether NT3 induces corticospinal sprouting directly or indirectly. Corticospinal axons express both trkC and the p75 ^NTR^ receptors ( Brock *et al.* , 2010 ) so it is plausible that if NT3 is secreted after transport to the cervical cord ( von Bartheld *et al.* , 1996 ; Altar and DiStefano, 1998 ) it may serve as a chemoattractant and cause spared corticospinal axons to form new connections or strengthen existing ones. We and others have already shown that direct application of NT3 to the CNS encourages growth of spared corticospinal fibres following injury ( Schnell *et al.* , 1994 ; von Meyenburg *et al.* , 1998 ; Zhou *et al.* , 2003 ; Chen *et al.* , 2006 ). Others have shown that radiolabelled NT3 can cross the intact mouse blood–brain barrier to enter the brain and cervical spinal cord ( Poduslo and Curran, 1996 ; Pan *et al.* , 1998 ; Pan and Kastin, 1999 ). We predict that higher levels of NT3 enter the brain and spinal cord after stroke when the blood–brain barrier is disrupted.

NT3 might also induce corticospinal sprouting indirectly, perhaps by causing the synthesis and secretion of other molecules by dorsal root ganglion neurons or by motor neurons. Dorsal root ganglion neurons make and secrete BDNF and in turn, and BDNF can cause corticospinal sprouting in rodent models of CNS injury ( Ueno *et al.* , 2012 ). Moreover, NT3 can cause proprioceptive dorsal root ganglion neurons to synthesize and secrete IGF1 ( Lee *et al.* , 2012 ) and antibodies against IGF1 can disrupt corticospinal sprouting in development ( Ozdinler and Macklis, 2006 ). It is likely that NT3 also improved sensorimotor function via spinal locomotor reflex circuits between proprioceptors and motor neurons ( Taylor *et al.* , 2001 ; Chen *et al.* , 2002 ; Patel *et al.* , 2003 ; Arvanian *et al.* , 2006 ; Petruska *et al.* , 2010 ; Takeoka *et al.* , 2014 ): indeed, one of us has previously shown that intramuscular injection of AAV1-hNT3 can improve locomotor function after spinal cord transection (i.e. in the absence of supraspinal input) by modifying proprioceptive circuits) ( Petruska *et al.* , 2007 ). In the present study we confirm that NT3 protein levels are elevated in the dorsal root ganglia ipsilateral to the muscles injected with AAV1-hNT3; in future experiments we will determine whether NT3 modifies proprioceptive locomotor reflexes. Finally, other descending tracts including the serotonergic raphe-spinal tract express NT3 receptors ( Arvidsson *et al.* , 1994 ); sprouting in multiple descending pathways may contribute to the observed functional recovery.

In conclusion, our study has shown that AAV1-hNT3 therapy enhances functional recovery in rats when initiated 24 h after stroke. This is clinically feasible because the median time to hospital admission and diagnosis is >6 h in major capital cities ( Evenson *et al.* , 2009 ). Furthermore, the therapy involves the human *NTF3* transgene, which works when administered in a clinically straightforward route to disabled muscles. It has already been shown in humans that AAV1 is safe and leads to muscle expression (of another transgene) for at least 1 year and high peripheral doses of recombinant NT3 have been shown to be safe in phase I and II clinical trials ( Chaudhry *et al.* , 2000 ; Coulie *et al.* , 2000 ; Parkman *et al.* , 2003 ; Sahenk, 2007 ). Thus, NT3 joins rehabilitation and antibodies against Nogo-A as a therapy that can restore motor function after stroke in an elderly nervous system when treatment is delayed by more than a few hours.

## Supplementary Material

Supplementary Data

## Abbreviations

AAV: adeno-associated virus
BDA: biotinylated dextran amine
NT3: neurotrophin-3
