# Supplementary material for: Delayed intramuscular human neurotrophin-3 improves recovery in adult and elderly rats after stroke
Source: Brain. 2015 Nov 27;139(1):259–75. doi: 10.1093/brain/awv341 (PMC4785394; doi:10.1093/brain/awv341)
Supplement: Supplementary Data [file awv341_supplementary_data.zip › brain-2015-00218-File012.pdf]

Supplementary Figure 1: Functional brain imaging during stimulation of the affected wrist revealed no enhanced probability of perilesional activation by neurotrophin-3. Elderly rats were imaged eight weeks after stroke and intramuscular treatment with either AAV-NT3 or AAV-EGFP. Heat map shows t-values obtained by Statistical Parametric Map analysis without correction for multiple testing ($p<0.05$). Red voxels denote greater probability of activation during stimulation in the NT3 group relative to the GFP group whereas blue voxels denote lesser probability of activation during stimulation in the NT3 group relative to the GFP group. There were no differences between the two groups for perilesional voxels and all other voxels did not pass the threshold for significance when corrected for multiple testing ($p<0.01$, data not shown because heat map was black).
