# Supplementary figures and images for: Delayed intramuscular human neurotrophin-3 improves recovery in adult and elderly rats after stroke
Source: Brain. 2015 Nov 27;139(1):259–75. doi: 10.1093/brain/awv341 (PMC4785394; doi:10.1093/brain/awv341)

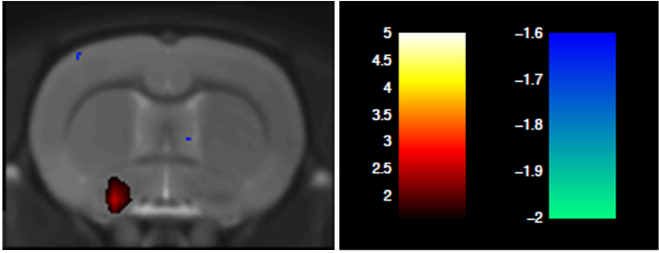

Supplement: Supplementary Data [file awv341_supplementary_data.zip › brain-2015-00218-File013.pdf]

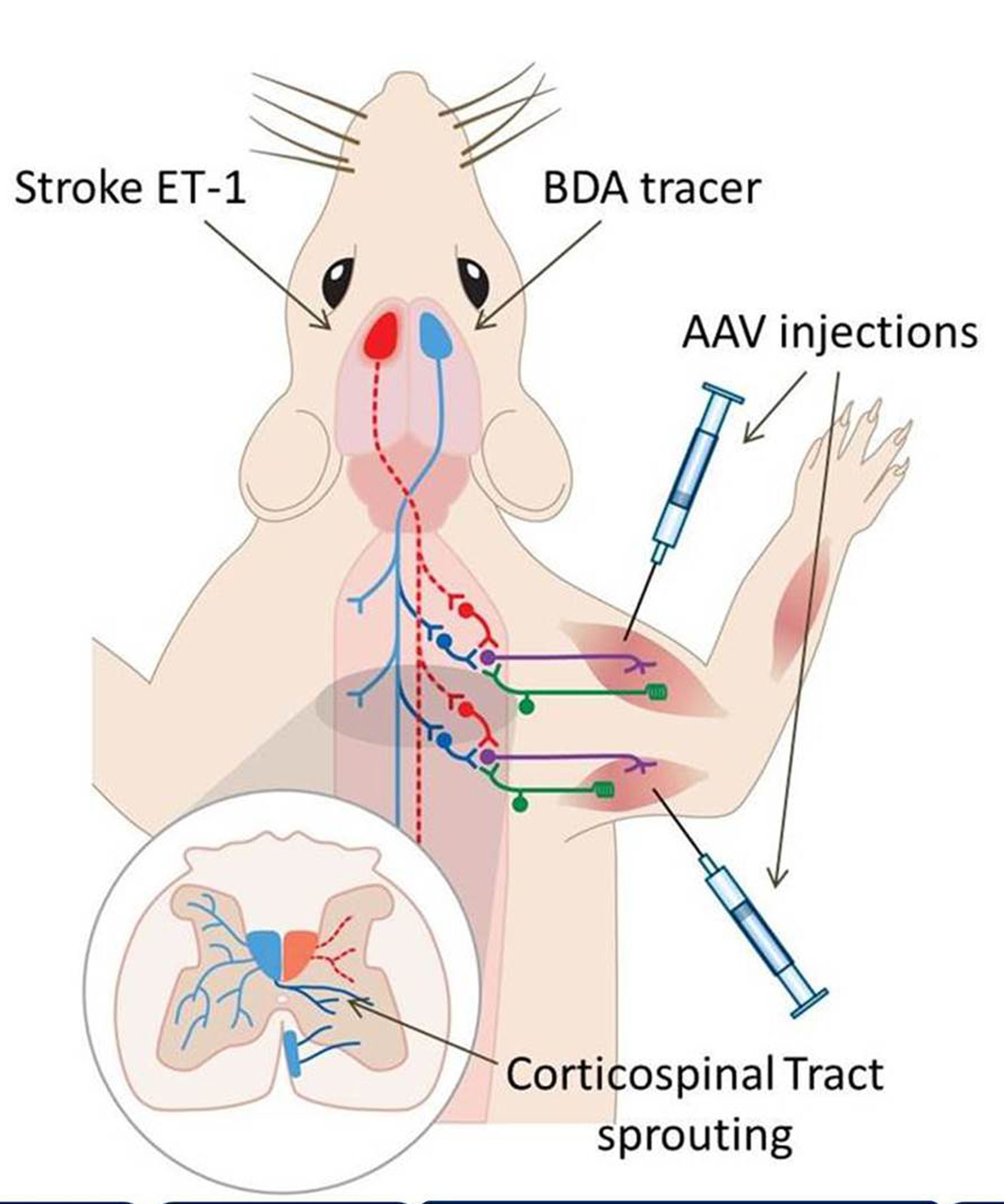

Supplement: Supplementary Data [file awv341_supplementary_data.zip › brain-2015-00218-File010.jpg]
